# Probiotic Supplementation Promotes Calcification in *Danio rerio* Larvae: A Molecular Study

**DOI:** 10.1371/journal.pone.0083155

**Published:** 2013-12-17

**Authors:** Francesca Maradonna, Giorgia Gioacchini, Silvia Falcinelli, Daniela Bertotto, Giuseppe Radaelli, Ike Olivotto, Oliana Carnevali

**Affiliations:** 1 Dipartimento di Scienze della Vita e dell'Ambiente, Università Politecnica delle Marche, Ancona, Italia; 2 Dipartimento di Biomedicina Comparata e Alimentazione, Università degli Studi di Padova, Legnaro (Padova), Italia; 3 Istituto Nazionale Biostrutture e Biosistemi, Roma, Italia; University of Texas Southwestern Medical Center, United States of America

## Abstract

A growing number of studies have been showing that dietary probiotics can exert beneficial health effects in both humans and animals. We previously demonstrated that dietary supplementation with *Lactobacillus rhamnosus* - a component of the human gut microflora - enhances reproduction, larval development, and the biomineralization process in *Danio rerio* (zebrafish). The aim of this study was to identify the pathways affected by *L. rhamnosus* during zebrafish larval development. Our morphological and histochemical findings show that *L. rhamnosus* accelerates bone deposition through stimulation of the expression of key genes involved in ossification, e.g. runt-related transcription factor 2 (*runx2*), Sp7 transcription factor (sp7), matrix Gla protein (*mgp*), and bone gamma-carboxyglutamate (gla) protein (*bglap*) as well as through inhibition of sclerostin (*sost*), a bone formation inhibitor. Western blot analysis of mitogen-activated protein kinase 1 and 3-(Mapk1 and Mapk3), which are involved in osteoblast and osteocyte differentiation, documented an increase in Mapk1 16 days post fertilization (dpf) and of Mapk3 23 dpf in individuals receiving *L. rhamnosus* supplementation. Interestingly, a reduction of *sost* detected in the same individuals suggests that the probiotic may help treat bone disorders.

## Introduction

Microbiota are endowed with the ability to modulate the transcription of hundreds of genes, including some of those involved in nutrient metabolism [1]. Lately, interest in the benefits of probiotic supplementation on teleost health [2], [3], immune function [4]–[8], stress tolerance [9] and development [10]–[13] has significantly augmented. Considerable attention has also been devoted to the effects of these bacteria on reproduction; indeed we found in a zebrafish model that the probiotic *Lactobacillus rhamnosus* affects the endocrine control of the hypothalamus–pituitary-gonadal axis by stimulating follicle maturation and inhibiting apoptotic processes naturally occurring in the ovary, thus enhancing fecundity [14]–[18]. Recently, Avella and co-workers [12] documented its ability to accelerate zebrafish backbone calcification and gonad differentiation by acting on GnRH and IGF systems; they also demonstrated that chronic administration of *L. rhamnosus* may influence the microbioma and in turn the host's development, opening new prospects for probiotic use and their applications. The above findings prompted us to examine the effects of *L. rhamnosus* on skeletal development, in particular on the modulation of the key genes responsible for osteoblastogenesis. In this process a delicate interplay of developmental cues, protein signaling, transcription factors and their regulators supports the differentiation of osteogenic lineage cells from the initial mesenchymal stem cell (MSC) to the mature osteocyte. In recent years zebrafish have increasingly been used as complements to traditional model organisms. Although, unlike other vertebrates including mouse and chicken, they have not often been employed as models to investigate bone disorders, the extensive conserved syntenic fragments with the human genome and the many structural and functional gene similarities prompted us to use them to investigate the effects of probiotic supplementation on the expression of some key signals involved in mammalian bone formation. In fact, the cells involved in zebrafish bone formation and remodeling are similar under many respects to those of mammals [19], [20], with osteoblasts and both mononucleated and multinucleated osteoclasts [21]. Despite the good conservation of the basic types of skeletal tissue and of the transcription factors, signaling molecules and hormones responsible for skeletal cell differentiation and skeletal development among vertebrates [22], [19], the mammalian and teleost skeleton differ considerably [23]. Some important differences found in teleosts, including zebrafish, involve i) the persistence of a cartilage rod inside a bone tube, with cartilage protruding as a condyle; in case of loss, cartilage is replaced by adipose tissue, and hematopoiesis occurs in the head kidney [21], [24]; ii) the existence of at least five types of cartilage [25], [26] ranging from cartilage-like connective tissue to bone-like cartilage [24]; iii) the presence of acellular mineralized tissue, the notochord sheath [27]; iv) the development of vertebral bodies without cartilaginous precursors - hence remodeling - which in mammals occurs by endochondral ossification [27]; and v) bone resorption, which in advanced teleosts relies on small, mononucleated osteoblasts [20].

In mammals, the master genes of osteoblast differentiation are *Runx2* and *Sp7*. R*unx2*, expressed in early osteoprogenitors, induces the gene expression program required for MSC lineage determination and differentiation and is also required for osteoblast function after differentiation. Although an important role for *Runx2* in the osteoblast phenotype has clearly been established, the gene is not osteoblast-specific, since it is expressed in the early development stages of numerous cell types, e.g. chondrocytes [28], [29]. Two *runx2* orthologs have been identified and characterized in zebrafish [30], [31]; *runx2a* and *runx2b* are both expressed in developing skeletal elements and show differences in their expression patterns. Defects are found following down-regulation of either gene [32]; moreover *runx2b*, whose expression is closely regulated by *twist* (which in turn lays the foundations for the dorso-ventral patterning [33]), is directly involved in regulating *sp7* and *bglap* [34], [35].

*Sp7* is among the few characterized osteoblast-specific genes and is thought to be involved in the regulation of numerous osteoblast genes including osteocalcin, osteonectin, osteopontin, bone sialoprotein and collagen type I [36], [37]. Knockout of mouse *Sp7* results in complete absence of ossification and osteoblasts, despite the presence of partially differentiated MSC [38]. Interestingly, S*p7−/−* mice do not exhibit altered *Runx2* levels, suggesting that *Sp7* likely acts downstream or independently of *Runx2* [38], [39]. BGLAP or osteocalcin and MGP, of the family of Ca ^2+^-binding vitamin K-dependent proteins, also have a major role in calcium metabolism and skeletal development. Molecular studies have demonstrated that BGLAP accumulates in the extracellular matrix of mammalian mineralized bone and its expression is specific to bone tissue and dentin [40], whereas MGP is mainly associated with cartilage, soft tissue and the vascular system [41]. In teleosts Bglap localizes to all mineralized tissues, including bone and calcified cartilage, during and after calcification [42], whereas the non-structural protein Mgp has a more widespread tissue distribution [43].

Although its molecular mechanism of action is unclear, the available evidence indicates that *Mgp* inhibits mineralization [44].

Recently, sclerostin (*sost*-related protein) has been found to have a role in cartilage and bone formation during zebrafish embryo development [45]. The role of sclerostin in the pathogenesis of sclerosis and in the onset of bone disease has been intensively investigated over the past few decades. Its critical negative role as a regulator of bone formation in the aging skeleton suggested that antibody-mediated inhibition of sclerostin could be a promising new therapeutic approach to the anabolic treatment of bone-related disorders, such as postmenopausal osteoporosis [46].

In addition, osteoblasts respond to and differentiate as a consequence of two main factors: chemical signals and physical stress. These stimuli activate specific signaling pathways such as MAPK (mitogen-activated protein kinase), whose action culminates in MAPK activation by MEK –a link between mechanosensitive cell surface integrin interactions with the extracellular matrix–and *Runx2* activation [47]. Surprisingly, relatively little is known about the possible specific roles of the two major MAPK isoforms, MAPK3 (p44) and MAPK1 (p42). The two proteins are co-expressed in virtually all tissues, albeit with quite a variable relative abundance, MAPK1 being the predominant isoform in brain and hematopoietic cells [48]. Given their extensive amino acid identity and their ostensibly similar spatio-temporal regulation, they are considered as interchangeable in most studies. However, recent evidence suggests quantitative/qualitative differences in their functioning, MAPK1 being essential for signal transduction and MAPK3 possibly enabling fine-tuning of its activity [49]. Genetically engineered mouse models allowed demonstration that both play an essential role in osteoblast and chondrocyte differentiation [50].

In this study alcian blue/alizarin red double staining and von Kossa histochemical staining demonstrated that probiotic administration accelerates skeletal formation in *Danio rerio* (zebrafish) larvae, and real-time PCR documented changes in the expression of a set of key genes involved in bone metabolism.

## Materials and Methods

### Ethics statement

All procedures involving animals were conducted in accordance with the Italian law on animal experimentation and were approved by the Ethics Committee of Università Politecnica delle Marche. All efforts were made to minimize suffering and a humane endpoint was applied with an excess of anesthetic (MS222, Sigma-Aldrich, Milano, Italy) when animals reached a moribund state.

### Animals and probiotic administration

Adult female and male zebrafish specimens purchased from a local supplier (Acquario di Bologna, Bologna, Italy) were acclimated to the laboratory conditions and their health state was monitored for 4 weeks prior to beginning the experiments. Pairs were spawned individually; larvae were kept in a 12∶12 h light/dark cycle at 27°C, grown to approximately 6 months of age, and used as breeders. Embryos were collected and divided into a control group (C), which was fed a commercial diet, and a probiotic-treated group (P), which received a commercial diet containing lyophilized *L. rhamnosus* IMC 501® (C025396A; Synbiotec, Camerino, Italy) at a concentration of 10^9^ colony-forming units (CFU)/g, to provide 10^6^ CFU/larva/day from hatching (2days post fertilization) to 21 days post hatching (dph), 23 days post fertilization (dpf), the skeletal mineralization period. The two groups comprised 200 larvae each. The experiment was set up in triplicates, with 3 control tanks and 3 probiotic tanks. Each replicate control (C) and probiotic (P) was generated by a different zebrafish pair. Sample larvae were collected in triplicate from each tank at 9,16, and 23 dpf at 08:00 am, to obtain 9 replicates for C and 9 for P.

Larvae were then anesthetized (0.1 g l^−1)^ using MS222 (Sigma-Aldrich) and stored at −80°C for real-time PCR and Western blot analysis or fixed in 4% paraformaldehyde (PFA) for von Kossa and alcian blue-alizarin red double staining.

### Morphological studies

At each time point larval length was measured using a Stemi 2000 micrometric microscope (Zeiss Vision Italia, Castiglione Orona, Italy) and weight was determined to an accuracy of 0.10 mg using a Microbalance (OHAUS Explorer E11140, Pine Brook, NJ, USA).

### Acid-free double-staining protocol

Double staining was performed according to Walker and Kimmel [51]. Acid-free double stain solution envisages two parts that are mixed just prior to staining: Part A is alcian blue 8 GX (C.I. 58005) for cartilage staining and Part B is alizarin red S (C.I. 74240, both from Sigma, St. Louis, MO, USA) for bone staining. Part A was obtained by first making a stock of 0.4% alcian blue in 70% ethanol; final concentrations were 0.02% alcian blue, 100 mM MgCl2, and 70% ethanol. Ouracid-free solution contained 10 ml Part B and 1 ml Part A.

### Tissue fixation

At 9, 16, 23 dph larvae were anesthetized with tricaine and fixed in 4% PFA in phosphate buffered saline (PBS) [52]. After rocking at room temperature for 2 h, the fixative was removed and larvae were washed and dehydrated with 1 ml ethanol 50% with rocking, at room temperature for 10 min.

#### Staining

After removing the ethanol, 1 ml acid-free double stain solution was added to the larvae and rocked at room temperature overnight.

#### Bleaching

Pigmentation was removed with a bleach solution made just before use by mixing equal volumes of 3% H_2_O_2_ and 2% KOH to a final concentration of 1.5% H_2_0_2_ and 1% KOH; 1 ml bleach solution was added and the tubes were sat with the lids open at room temperature for 20 min.

#### Clearing

Tissues were cleared with successive changes of a 20% glycerol and 0.25% KOH solution and rocked at room temperature for 30 min to overnight. The solution was replaced with 1 ml 50% glycerol and 0.25% KOH and rocked at room temperature for 2 h to overnight. Larvae were viewed in the same solution under the Stemi 2000 micrometric microscope, stored in a solution of 50% glycerol and 0.1% KOH at 4°C and photographed with a Zeiss Axioskop equipped with a digital camera.

### von Kossa staining protocol for calcium

Larvae for this protocol were collected at 9, 16, 23 dpf, anesthetized with tricaine, fixed in 4% PFA prepared in PBS 0.1 M, pH 7.4 overnight at 4°C, washed in PBS, dehydrated through a graded series of ethanol and embedded in paraffin. Consecutive sections 4 µm in thickness were cut using a microtome, deparaffinized in toluene and hydrated by serial washing in graded ethanol and distilled water. Finally, von Kossa staining for mineral deposits was performed with a fast red counterstain [53].Mineral deposits were visualized as black areas.

### RNA extraction and cDNA synthesis

Total RNA was extracted from 15 whole larval bodies using RNAeasy® Minikit (Qiagen, Milano, Italy) following the manufacturer's instructions; it was then eluted in 15 µl of RNAse-free water. Final RNA concentrations were determined using the Nanophotometer ™P-Class (Implem GmbH, Munich, Germany); RNA integrity was verified by ethidium bromide staining of 28S and 18S ribosomal RNA bands on 1% agarose gel. RNA was stored at −80°C until use. Total RNA was treated with DNAse (10 IU at 37°C for 10 min, MBI Fermentas, Milano, Italy). A total amount of 1 µg RNA was used for cDNA synthesis with iScript cDNA Synthesis Kit (Bio-Rad, Milano, Italy).

### Real-time PCR

PCRs were performed with SYBR green method in an iQ5 iCycler thermal cycler (Bio-Rad) in triplicate. The reactions were set up on a 96-well plate by mixing, for each sample, 1 µl of diluted (1/20) cDNA, 5 µl of 2X concentrated iQ TM SYBR Green Supermix (Bio-Rad), containing SYBR Green as a fluorescent intercalating agent, 0.3 µM of the forward primer and 0.3 µM of the reverse primer. The thermal profile for all reactions was 3 min at 95°C followed by 45 cycles of 20 sec at 95°C, 20 sec at 60°C and 20 sec at 72°C. Fluorescence was monitored at the end of each cycle. Dissociation curve analysis showed a single peak in all cases.

*β-actin* (*actb*) and *acidic ribosomal protein* (*rplp*) were used as the housekeeping genes to standardize the results by eliminating variation in mRNA and cDNA quantity and quality. No amplification product was observed in negative controls and primer-dimer formation was never seen in control templates. Data were analyzed using Bio-Rad's iQ5 optical system software, version 2.0. Modification of gene expression is reported with respect to the control sample. Primer sequences for *runx2*, *sp7*, *sost*, *actb* and *rplp* were designed using Primer3 (210 v. 0.4.0); primers for *mgp* and *bglap* were from [42]. All primers are listed in Table 1. The *runx2* primer set was designed in a high-homology region for the *runx2a* and *b* orthologs.

**Table 1:** Primer list.

Gene	Forward	Reverse
*runx2*	5′-CTCCTCAGACCGGAGCGCGT-3′	5′-GGCTGAAGGCTGCTGGACGG-3′
*sp7*	5′-AACCCAAGCCCGTCCCGACA-3′	5′-CCGTACACCTTCCCGCAGCC-3′
*mgp* [42]	5′-AACACAACCCCTACATCTACCGAA-3′	5′-GCGGGCTGAAGAAGGTCTGATAGG-3′
*bglap* [42]	5′-GCCTGATGACTGTGTGTCTGAGCG-3′	5′-AGTTCCAGCCCTCTTCTGTCTCAT-3′
*sost*	5′-ACAATGAATCGGGCGAAGAA-3′	5′-GTTCTGAGGCTCCATAAGTCC-3′
*rplp*	5′-CTGAACATCTCGCCCTTCTC-3′	5′-TAGCCGATCTGCAGACACAC-3′
*actb*	5′-GGTACCCATCTCCTGCTCCAA-3′	5′-GAGCGTGGCTACTCCTTCAC -3′

### Western blotting

For the Mapk1/3 assay, whole larval homogenates from 10 larvae per group were electrophoresed and transferred to PVDF [54]. Briefly, 20 µg of each protein sample was separated using 4% stacking and 10% separating sodium dodecyl sulfate polyacrylamide gel electrophoresis (SDS-PAGE) [55], and electroblotted onto a filter using a mini trans-blot electrophoretic transfer cell (all from Bio-Rad). Transfer was carried out for 30 min using Bio-Rad's Trans-Blot® Turbo™ Transfer System.

The membrane was soaked in 5% Nonidet-P40 for 1 h to remove SDS and incubated with 2% bovine serum albumin (BSA; Sigma) in PBS. The Mapk1/3 primary antibody (sc-292838; Santa Cruz Biotechnology, Santa Cruz, CA, USA) was diluted 1∶1000 in a solution containing 2% BSA, 0.01% NaN3 in PBS, incubated for 2 h at room temperature (about 20°C), and rinsed 3 times with PBS plus 0.05% Tween 20. α-tubulin (Tuba1b), diluted 1∶1000 was used as the internal standard (H-300 sc5546 Santa Cruz Biotechnology). The reaction was visualized with ECL-PLUS (GE Healthcare, Milano, Italy) chemiluminescent reagent for Western blotting. Densitometric analysis was performed using ImageJ software for Windows.

### Statistical analysis

One-way analysis of variance followed by Bonferroni's multiple comparison test was used to determine differences among groups. Statistical significance was set at P<0.05.

## Results

### Morphometry findings

At each sampling time larvae supplemented with *L. rhamnosus* had a greater body weight. No significant length differences were detected between C and P larvae at 9 dpf, whereas at 16 and 23 dpf the larvae receiving the probiotic were significantly longer than control individuals (P<0.05) (Table 2).

**Table 2:** Total length and wet weight.

	Total length TL (mm)		Wet weight WW (mg)	
	C	P	C	P
**9 dpf**	3.86±0.26	4.45±0.32*	1.17±0.17	1.63±0.12
**16 dpf**	4.87±0.26	5.5±0.27*	2.41±0.08	3.21±0.23*
**23dpf**	8.72±0.48	9.82±0.58*	7.16±0.29	8.25±0.23*

Total length (TL) and wet weight (WW) of zebrafish larvae from control (n = 15) and probiotic (n = 15) groups, collected at 9, 16 and 23 days post fertilization (3 replicates). Asterisks indicate significant differences (P<0.05).

### Zebrafish skeletal development: alcian blue-alizarin red double staining

**Figure 1:**
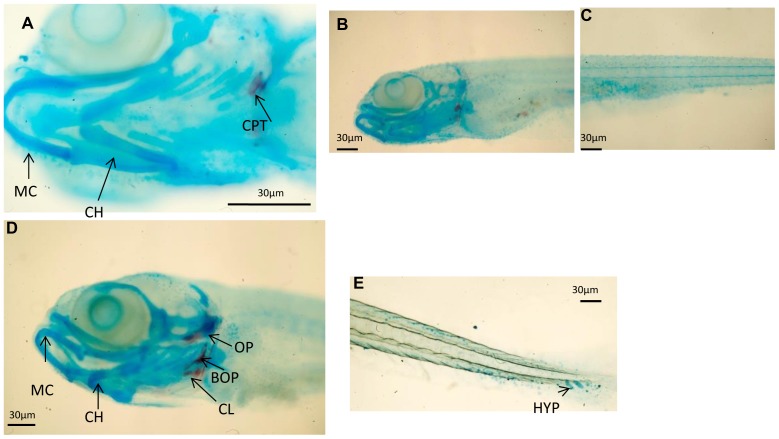
Skeletal development in zebrafish using alcian blue-Alizarin red double staining. A 9 dpf zebrafish control larvae head skeleton presenting calcified calcified pharyngeal teeth (CPT)while other structures like Meckel's cartilage (MC) and ceratohyal (CH) remain as cartilage); (B–C) 9 dpf control zebrafish head (B) and trunk (C) presenting no signals of bone calcification.(A) A 9 dpf *L. rhamnosus* fed zebrafish larvae head skeleton presenting calcification of the opercula (OP), cleithrum (CL) and basioccipital articulatory process (BOP). Meckel's cartilage (MC) and ceratohyal (CH) remain as cartilage; (E) 9 dpf *L. rhamnosus* fed zebrafish presenting the first hypurals (HYP) developing. Scale bar: 30 µm.

**Figure 2:**
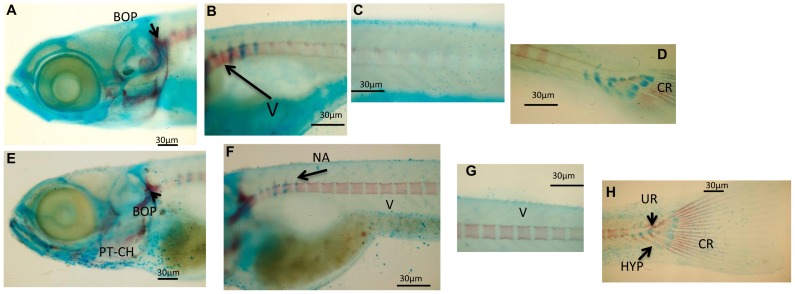
Whole mounts double staining of the skeleton in larvae sampled at 16 dpf. (A–D) Images showing significant aspects of skeleton development in control zebrafish larvae. (B) Formation of first vertebrae (V). (D) Caudal hypuralia aquires final number of structures with modified hemal arches (MHA) and caudal fin rays (CR). (E–H) representative images showing the development of the skeleton in zebrafish fed *L. rhamnosus*. (E) presence of calcified pharyngeal teeth (PT) and ceratohyal (CH). (F–G) Vertebrae formation (in an anterior-posterior direction) toward the posterior end of the notochord. Formation of the first neural arches (NA) is observed dorsally in the anterior vertebrae. (H) Beginning of calcification of the hypurals (HYP) under the urostyle (UR) and presence of calcified caudal fin rays. Scale bar: 30 µm.

**Figure 3:**
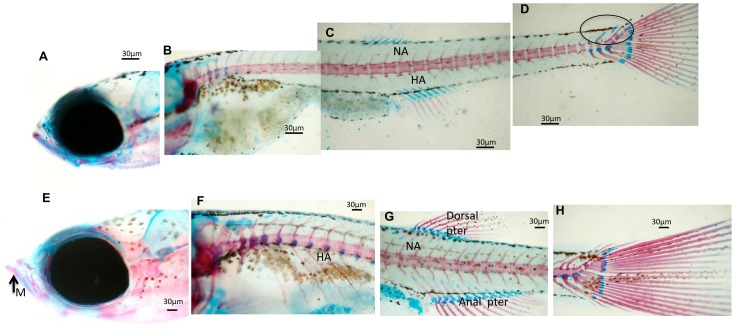
Whole mount double staining of the skeleton in larvae sampled at 23 dpf. (A–D) Images showing significant aspects of skeleton development in control zebrafish larvae. Neural arches (NA) and sketches of hemal arches (HA) are evidenced. Caudal skeleton still presents cartilaginous structures evidenced by a circle. (E–H) Representative images showing the development of the skeleton in zebrafish fed *L. rhamnosus*. (E) Presence of calcified mandibular (M). Neural arches (NA) and hemal arches (HA) are detected in the whole trunk of the larvae. (G) Complete formation of dorsal and anal pterygium.(H) Caudal skeleton is complete. Scale bar: 30 µm.

The ontogenetic development of cartilaginous and calcified structures was followed in double-stained whole-mount zebrafish preparations. At 9dpfcontrol larvae had calcified pharyngeal teeth (CPT) in the head skeleton, whereas structures such as Meckel's cartilage (MC) and ceratohyal (CH) were still cartilage (Figure 1A). However, neither the head (Figure1B) nor the trunk (Figure 1C) showed signs of calcification. At this time point, the head skeleton of probiotic-treated larvae exhibited calcified opercula, cleithrum and basioccipital articulatory process (BOP), whereas MC and CH were still cartilage (Figure 1D), and the first hypurals (HYP) were developing (Figure 1E). At16 dpf examination of C larvae (Figure 2A–D) showed that the first vertebrae were forming (Figure 2B) and the caudal hypurals reached the final number of structures with modified hemal arches (MHA) and caudal fin rays (CR) (Figure 2D). At16 dpf larvae (Figure 2E–H) had calcified PT-CH (Figure 2E) and vertebrae (in an anterior-posterior direction) in the posterior end of the notochord (Figure 2F–G); the first neural arches (NA) were forming dorsally in the anterior vertebrae, as were HYP calcification under the urostyle and caudal fin rays (Figure 2H). At 23 dpf (Figure 3A–D) C larvae showed mineralized NA and HA, while the caudal skeleton still exhibited cartilaginous structures. In P larvae (Figure 3E–H) the mandibular was calcified (Figure 3E) and NA and HA were detected in the whole trunk. The dorsal and anal pterygia were wholly formed (Figure 3G) and the caudal skeleton was complete (Figure 3H).

### von Kossa histochemical stain

**Figure 4:**
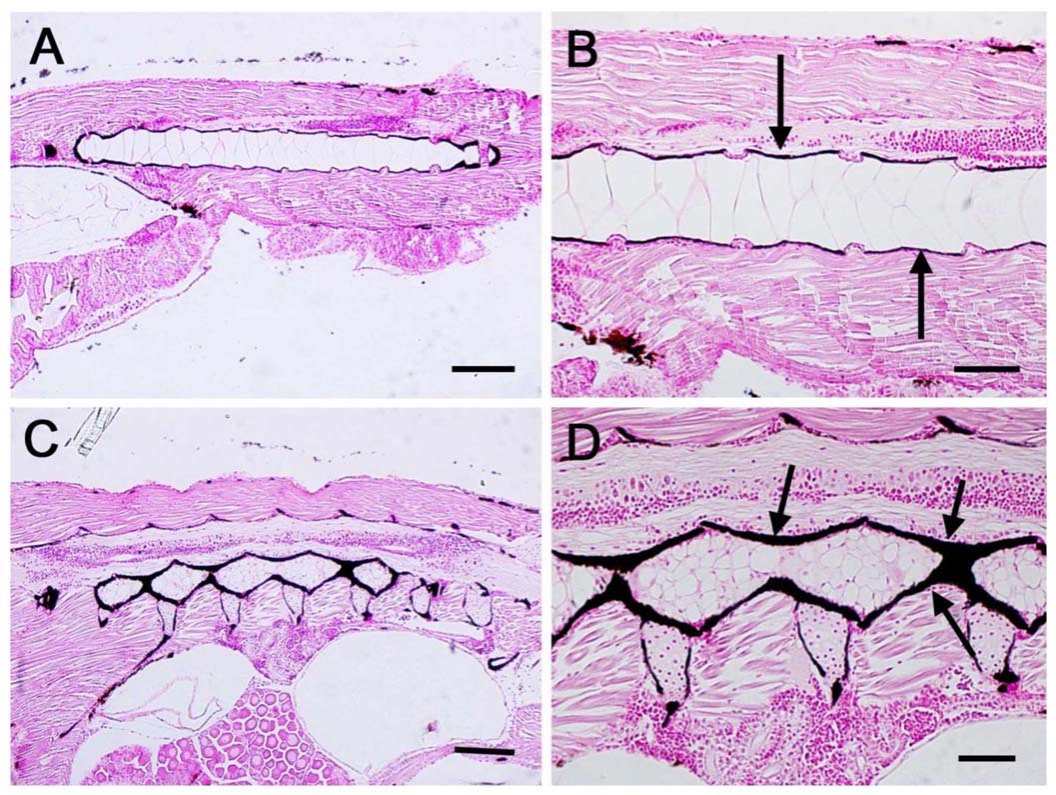
Von Kossa histochemical staining in larvae samples at 23 dpf. (A) Control zebrafish larva showing the mineral deposits around the notochord. (B) Higher magnification of image A. Arrows indicate the mineral deposits. (C) *L.rhamnosus* treated larva showing the mineral deposits around the vertebral bodies. (D) Higher magnification of image C. Arrows indicate the mineral deposits. Scale bars: A–C = 100 µm; B–D = 40 µm.

The von Kossa staining showed mineral deposits in the head skeleton and at the level of the notochord sheath. Black areas were seen already at 9 and 16 dpf, although no differences were apparent between C and P specimens. At 23 dpf the mineralization of vertebral bodies was much more evident in treated larvae (Figure 4A–D).

### Molecular findings

**Figure 5:**
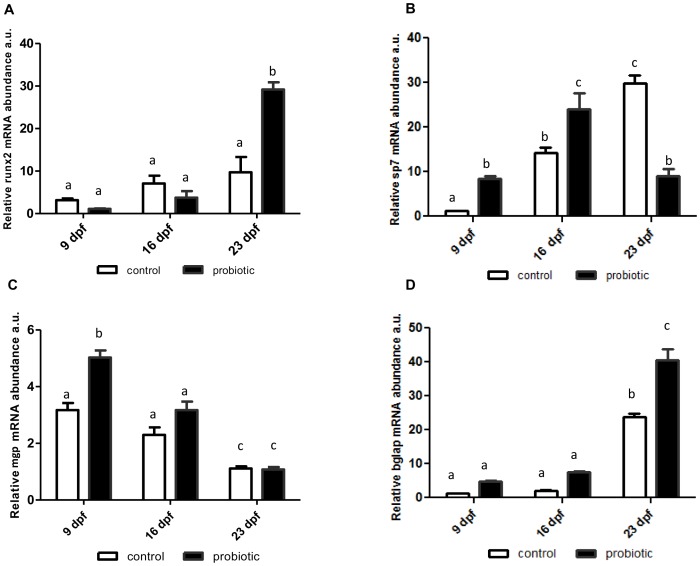
*runx2* (A), *sp7*(B), *mgp* (C), *bglap* (D) mRNA levels. mRNA levels normalized against *actb* and *rplp* in control and *L. rhamnosus* treated fingerlings sampled 9, 16 and 23 dpf. Error bars indicate mean ± S.D. Different letters denote statistical significant differences among experimental groups (p<0.05), analyzed using ANOVA followed by Bonferroni multiple comparison test.

**Figure 6:**
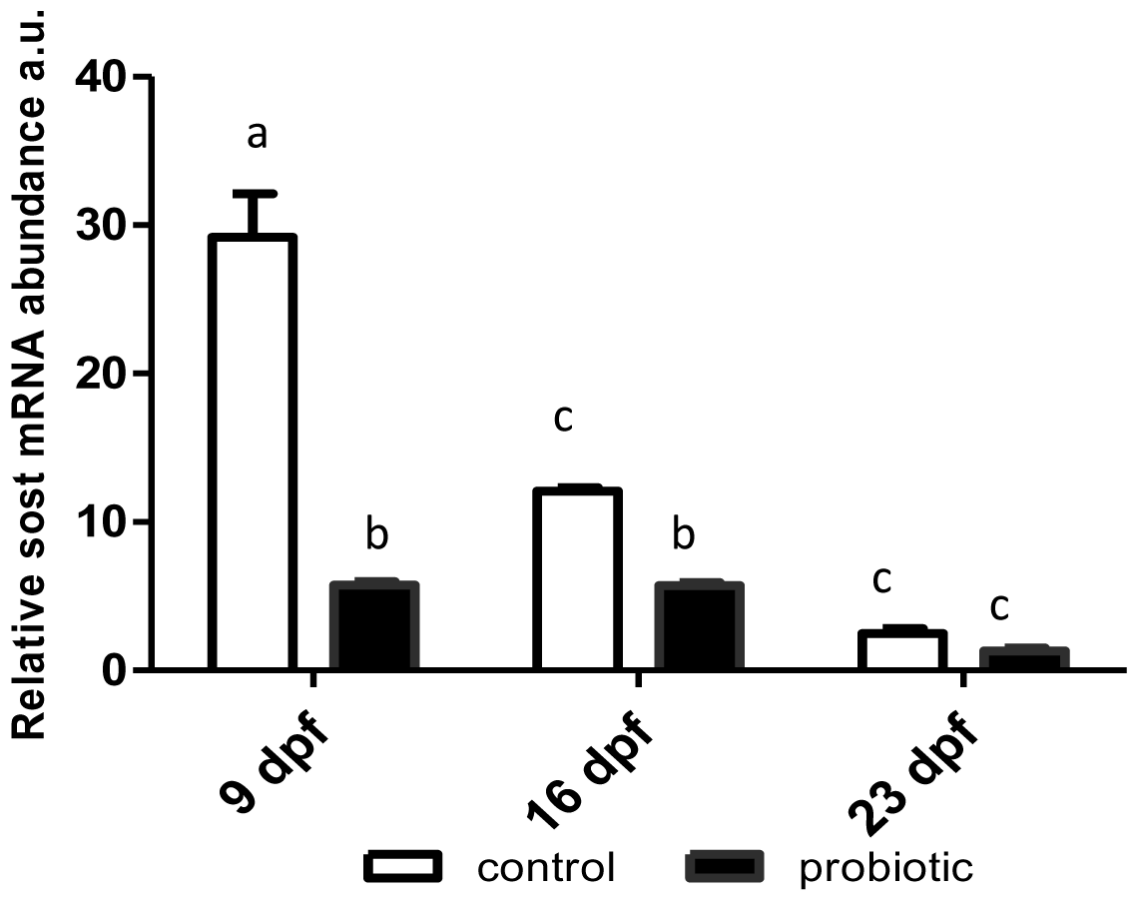
*sost* mRNA levels. *sost* mRNA levels normalized *actb and rplp* in control and *L.rhamnosus* treated fingerlings sampled 9,16and 23dpf. Error bars indicate mean ± S.D. Different letters denote statistical significant differences among experimental groups (p<0.05), analyzed using ANOVA followed by Bonferroni multiple comparison test.

**Figure 7:**
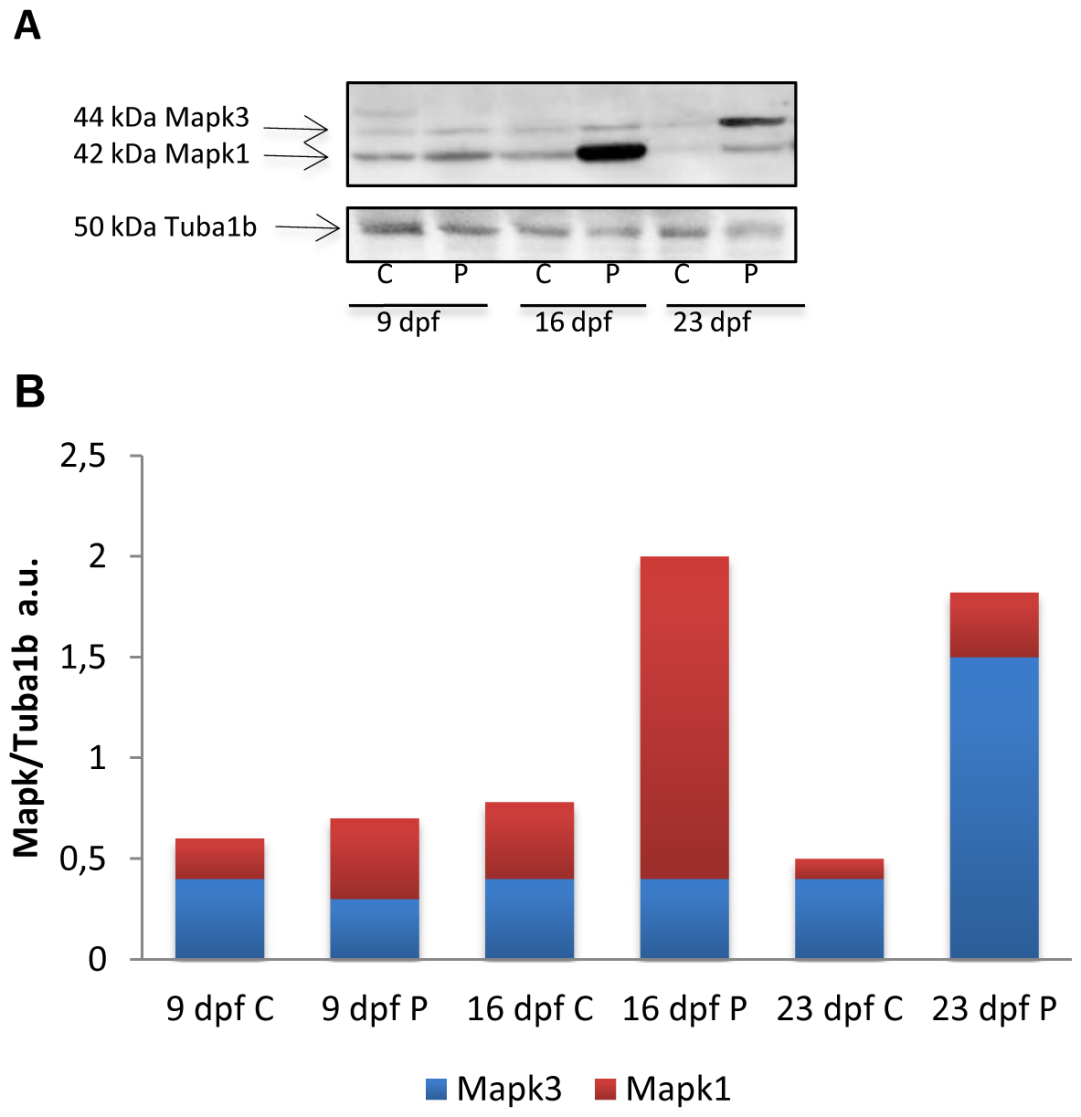
Mapk1/3 Western blot. A) Insert shows a representative Mapk1/3 and Tuba1b Western blot in control and *L. rhamnosus* treated fingerlings sampled 9, 16 and 23 dpf. C- control P-probiotic. B) Densitometric analysis of 3 independent experiments a.u. (arbitrary units). Statistical significant differences (P<0.05) were found for Mapk2 levels at 16 dpf P respect to control and for Mapk3 levels at 23 dpf P respect to control.

*runx2* gene expression did not vary significantly at the different time points in C larvae, whereas in P specimens its levels were significantly increased at 23 dpf compared with 9and 16 dpf and with C individuals (Figure 5A). *sp7* expression (Figure 5B) rose significantly in C larvae, showing at 23dpf a 30-fold increase compared with 9 dpf levels. In P larvae *sp7* expression peaked at 16 dpf and at 23 dpf reverted to 9 dpf levels(Figure 5B). Analysis of *mgp* gene transcripts showed lower levels at 23 dpf compared with 9 and 16 dpf in both groups; however levels were significantly higher in P vs. C zebrafish at 9 and 16 dpf and similar at 23 dpf (Figure 5C). *bglap* expression peaked at 23 dpf and was significantly greater in P specimens (Figure 5D). Peak *sost* expression was found at 9 dpf; it then declined, reaching the lowest level at 23 dpf. Interestingly, mRNA expression was higher in C larvae at 9and 16dpf, when probiotic treatment induced a significant sclerostin reduction, whereas at 23dpf there were no differences between C and P individuals (Figure 6).The Mapk 1/3 antibody cross-reacted with a doublet of the expected molecular weight (44–42 kDa) and showed similar levels in C and P specimens at 9dpf and an increase in Mapk1/3 protein levels at 16 and 23 dpf in P larvae; at 16 dpf the lower molecular weight component (Mapk1) increased, whereas the 44 kDa band (Mapk3) increased at 23 dpf (Figure 7A–B).

## Discussion

The findings of this study clearly demonstrate the beneficial effects of *L. rhamnosus* supplementation on zebrafish skeletal formation and confirm previous data from our laboratory [56]. Avella and co-workers [12] documented that the probiotic favorably influences zebrafish growth in terms of both weight and length, it up-regulates growth biomarkers (*igf1* and *igf2*) and down-regulates myostatin; calcein staining demonstrated a significantly increased centra/intercentra ratio in fish treated with *L. rhamnosus*, supporting their hypothesis that it is associated with higher rates of ossification. Based on these data we set out to study the effects of *L. rhamnosus* administration on ossification, also considering that these supplements are likely to find application in a variety of fields (including aquaculture) and possibly even in the treatment of bone homeostasis dysfunctions. To prevent the increased ossification from being attributed to a faster growth rate, we picked and tested specimens of similar size. The rate of malformations did not differ between the two groups.

Bone and cartilage metabolism and the mineralization process have elicited considerable interest over the past few years and efforts have been made to clone the genes involved. Recently, Lamari and co-workers [57] found in seabass, *Dicentrarchus labrax*, that Lactobacilli can exert a positive or negative action on skeletal formation. They demonstrated that *Pediococcus acidilactici* enhances the ossification rate while *Lactobacillus casei* increases the incidence of vertebral deformities. The contrasting effects seemed to be related to a different modulation of *bglap* by the two bacteria. These findings suggested that evaluation of the effects of probiotics on growth should also take into account the molecular signaling involved in skeletal development. The above data prompted us to combine morphometric evaluation and histochemical staining (von Kossa and alcian blue/alizarin red) with the analysis of the expression of *runx2* and *sp7*, the genes involved in early osteoblast differentiation and bone formation. We found that they were up-regulated in the treated group, suggesting enhanced bone formation in these animals. However, changes in *runx2* expression should be carefully evaluated, since its overexpression in mammals is associated with bone-metastatic cancers [58], whereas haploinsufficiency causes cleidocranial dysplasia [59]. As regards *sp7*, the probiotic-induced up-regulation observed in this study precedes the *runx2* peak; this suggests that alternative pathways are involved in *sp7* expression control, as previously described in mammals, where *Msx2* overexpression stimulates osteoblast differentiation and regulates the balance between osteoblastogenesis and adipogenesis [60], [61]. A similar mechanisms may also be hypothesized in zebrafish.

In addition, the expression of *mgp*, a biomarker of mature cartilage [62], [63],which in mammals plays a role as an inhibitor of mineralization, peaked at 9dpfand declined in older fish. Similar data have been found in seabream, where *mgp* gene expression appears around 2 dph and peaks at 9 dph, demonstrating a close association with the early growth phases [64]. The *mgp* up-regulation observed in probiotic-treated zebrafish confirms that these fish are in a more advanced stage of development compared with controls. As observed in the same experimental model by Pinto and collaborators [34], *mgp* gene expression and protein accumulation reflect the patterns of formation of cartilaginous and mineralized structures. The *mgp* levels described herein agree with previous findings showing a decline in its expression when skeletal structures are mineralized [64]. As regards *bglap*, which codes for osteocalcin and is directly activated by *runx2*, acts downstream of the bone formation cascade and is a biomarker of the post-proliferative phase, which coincides with osteoid formation [65]. In the present study, *bglap* was significantly more expressed in treated larvae and showed the same trend as *runx2*.

*S*ost expression was highest in control animals at 9 dpf and gradually declined until 23 dpf. S*ost* encodes sclerostin, a protein synthesized by osteocytes that can down-regulate osteoblast formation. Its inhibition results in increased bone production, leading to the notion that compounds that reduce its levels could be harnessed to treat osteoporosis and other skeletal disorders [66]. S*ost* inhibition by *L. rhamnosus* seen in this study documented for the first time the ability of functional food additives to modulate the transcription of genes involved in bone metabolism, providing basic knowledge for the future application of probiotics to the treatment of bone dysfunction. The present findings agree with previous data obtained with *Lactobacillus reuteri*, which was demonstrated to increase bone density. The beneficial effects of probiotics may be related to their ability to reduce the intestinal inflammation that causes loss of bone mass. These data also suggest that probiotic treatment could help reduce the bone loss in osteoporotic patients [67].

Finally, we documented Mapk1/3 modulation following *L.rhamnosus* supplementation. While their tissue distribution is substantially established [68], their specific roles are still poorly known. Several studies have shown that the ERK MAPK pathway plays an essential role in mediating fibroblast growth factor signaling in skeletal cells [69], [70]. In a mouse model, genetic inactivation of MAPK1/3 in undifferentiated mesenchymal cells, induced severe impairment of osteoblast differentiation and bone formation [71], [72], while their inhibition induced ectopic cartilage formation in mouse perichondrium [50], supporting their pivotal role in skeletal development.

Whereas numerous *in vivo* and *in vitro* studies have examined osteoblast differentiation mechanisms, relatively little is known about the transition from osteoblasts to osteocytes, the most abundant cell type in bone. In the present study Western blot analysis suggested the possible involvement of the two Mapk isoforms in *sp7*, *runx2* and *bglap* activation. Peak *sp7* expression was detected in treated larvae at 16 dpf, concomitant with the increase in Mapk1 expression. In addition, *runx2* and *bglap* peaked in the treated group at 23 dpf, concomitant with the increase in the 44 kDa Mapk3 band. Jun and colleagues [73] demonstrated that mammalian ERK/MAP kinase controls *Sp7* transcription by R*unx2*. MAPK1/3 also directly raises *Sp7* mRNA and protein levels and stability. However, the precise mechanism by which MAPK1/3 modulates *Sp7* is still being investigated [74]. Considering the role of MAPK1/3 in mammals, our findings suggest that the Mapk1/3 modulation seen in treated larvae may be related to the acceleration of bone formation by *L.rhamnosus*.

**Figure 8:**
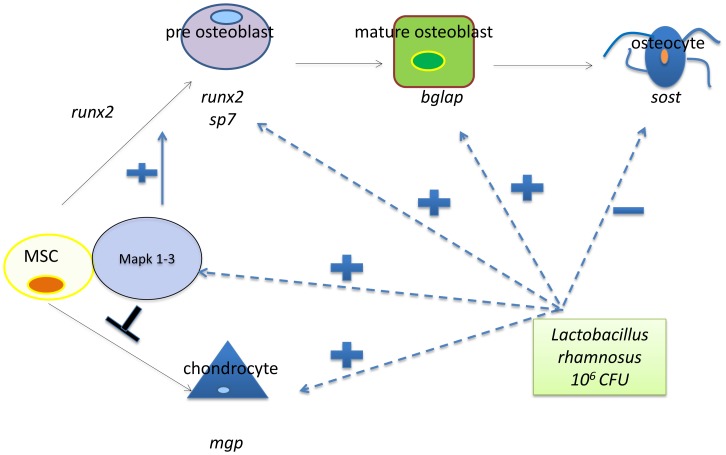
Proposed model of the role of *L. rhamnosus* in the regulation of osteoblast and chondrocyte differentiation.

In conclusion, our data document the positive effects of *L. rhamnosus* supplementation on skeletal development. At the molecular level, the treated group had higher levels of Mapk1/3, which may participate in the regulation of genes involved in osteocyte formation (see Figure 8). The evidence described here could be a starting point to gaining insights into normal bone homeostasis and the pathogenesis of conditions involving bone loss and skeletal deformities such as osteoporosis.

Since zebrafish have been established as a vertebrate model for biomedical research, the present findings provide data for the use of *L. rhamnosus* as a support to human treatment.
